# Nature's role in sustaining economic development

**DOI:** 10.1098/rstb.2009.0231

**Published:** 2010-01-12

**Authors:** Partha Dasgupta

**Affiliations:** 1 Faculty of Economics, University of Cambridge, Sidgwick Avenue, Cambridge CB3 9DD, UK; 2 Sustainable Consumption Institute, University of Manchester, 188 Waterloo Place, Oxford Road, Manchester M13 9PL, UK

**Keywords:** natural capital, human capital, population growth, shadow prices, comprehensive wealth, property rights

## Abstract

In this paper, I formalize the idea of sustainable development in terms of intergenerational well-being. I then sketch an argument that has recently been put forward formally to demonstrate that intergenerational well-being increases over time if and only if a comprehensive measure of wealth *per capita* increases. The measure of wealth includes not only manufactured capital, knowledge and human capital (education and health), but also natural capital (e.g. ecosystems). I show that a country's comprehensive wealth *per capita* can decline even while gross domestic product (GDP) *per capita* increases and the UN Human Development Index records an improvement. I then use some rough and ready data from the world's poorest countries and regions to show that during the period 1970–2000 wealth *per capita* declined in South Asia and sub-Saharan Africa, even though the Human Development Index (HDI) showed an improvement everywhere and GDP *per capita* increased in all places (except in sub-Saharan Africa, where there was a slight decline). I conclude that, as none of the development indicators currently in use is able to reveal whether development has been, or is expected to be, sustainable, national statistical offices and international organizations should now routinely estimate the (comprehensive) wealth of nations.

## 1. Questions and responses

Are humanity's dealings with nature sustainable? Can we expect world economic growth to continue in the foreseeable future? Should we be confident that knowledge and skills will increase in such ways as to lessen our reliance on nature in relation to humanity's growing numbers and rising economic activity?

Contemporary discussions on these questions are now several decades old. If they have remained alive and continue to be shrill, it is because two opposing empirical perspectives shape them. On the one hand, if we look at specific examples of what economists call *natural capital* (aquifers, ocean fisheries, tropical forests, estuaries, the atmosphere as a carbon sink—ecosystems, generally), there is convincing evidence that at the rates at which we currently exploit them they are very likely to change character dramatically for the worse, with little advance notice. Indeed, many ecosystems have already collapsed, with short notice (M.E.A. 2003; Hassan *et al*. 2005). On the other hand, if we study historical trends in the prices of marketed resources (e.g. minerals and ores), or improvements in life expectancy, or growth in recorded incomes in regions that are currently rich and in those that are on the way to becoming rich, resource scarcities would not appear to have bitten. Suppose you were to point to the troubled nations of sub-Saharan Africa and suggest that resource scarcities are acute there today. Those with the former perspective (ecologists generally) will tell you that it is because people in the world's poorest regions face acute resource scarcities relative to their numbers that they are so poor, while those with the latter perspective (economists usually) will inform you that people there experience serious resource scarcities because they are poor. When experts disagree over such a fundamental matter as the direction of causation, there is little to go on.

Those conflicting intuitions are also not unrelated to an intellectual tension between the concerns people share about carbon emissions and acid rains that sweep across regions, nations and continents and about declines in the availability of firewood, fresh water, coastal resources and forest products in as small a locality as a village in a poor country. That is why ‘environmental problems’ present themselves in different ways to different people. Some identify environmental problems with population growth, while others identify them with wrong sorts of economic growth. There are those who identify environmental problems with urban pollution in emerging economies, while others view them through the spectacle of poverty. Each of those visions is correct. There is not just one environmental problem. There is a large collection of them, and they manifest themselves at different spatial scales and operate at different speeds (Ehrlich & Ehrlich 1981, 1990; Dasgupta 1993, 2001; Sachs 2008). In this reckoning, environmental pollutants are the reverse of natural resources. Roughly speaking, ‘resources’ are ‘goods’ (many being sinks into which pollutants are discharged); while ‘pollutants’ (the degrader of resources) are ‘bads’. Pollution is the other side of conservation. That is why pollution and conservation can be studied in a unified way (Dasgupta 1982).

Despite the conflicting intuitions, most economists would appear to be convinced that scientific and technological advances, the accumulation of reproducible capital (machinery, equipments, buildings and roads), growth in human capital (health, education and skills) and improvements in the economy's institutions (which are also capital assets) can overcome diminutions in natural capital. Otherwise, it is hard to explain why twentieth-century economics has been so detached from the environmental sciences. Judging by the profession's writings, we economists see nature, when we see it at all, as a backdrop from which resources and services can be drawn in isolation. Macroeconomic forecasts routinely exclude natural capital. Accounting for nature, if it comes into the calculus at all, is usually an afterthought to the real business of ‘doing economics’. We economists have been so successful in this enterprise, that if someone exclaims, ‘Economic growth!’, no one needs to ask, ‘Growth in what?’—we all know they mean growth in gross domestic product (GDP).

The rogue word in GDP is ‘gross’. Since GDP is the total value of the final goods and services an economy produces, it does not deduct the depreciation of capital that accompanies production—in particular, it does not deduct the depreciation of natural capital. In the quantitative models that appear in leading economics journals and textbooks, nature is taken to be a fixed, indestructible factor of production. The problem with the assumption is that it is wrong: nature consists of degradable resources. Agricultural land, forests, watersheds, fisheries, fresh water sources, river estuaries and the atmosphere are capital assets that are self-regenerative, but suffer from depletion or deterioration when they are over-used. (I am excluding oil and natural gas, which are at the limiting end of self-regenerative resources.) To assume away the physical depreciation of capital assets is to draw a wrong picture of future production and consumption possibilities that are open to a society.

Here is an illustration of what goes wrong in economic accounts when depreciation is ignored. Repetto *et al*. (1989) and Vincent *et al*. (1997) estimated the decline in forest cover in Indonesia and Malaysia, respectively. They found that when depreciation is included, national accounts look quite different: net domestic saving rates are some 20–30% lower than recorded saving rates. In their work on the depreciation of natural resources in Costa Rica, Solorzano *et al*. (1991) found that the depreciation of three resources (forests, soil and fisheries) amounted to about 10 per cent of GDP and over one-third of domestic saving.

## 2. Plan of the paper

In this paper, I want to give you a sense of how economics can be reconstructed to include natural capital in a seamless way. I shall do that in three stages. In §3, I show that property rights to natural capital are frequently unprotected or ill-specified. I argue that this typically leads to their overexploitation, and so to waste and inequity. In §4, I illustrate overexploitation in the context of a ‘small’ problem: the economic failure that can accompany deforestation in a small region. It will not require any stretch of imagination to recognize that every economy faces innumerable such ‘small’ problems. The performance of the macro-economy depends, of course, on how each of those small problems is tackled there. If good polices are in place to reduce the economic losses that are generated by the small problems, the macro-economy can be expected to function well; but not otherwise. So in §5, I demonstrate that when natural capital is included in economic statistics, the recent economic history of nations looks very different from what we are led to believe when conventional economic indicators, such as GDP per head or the United Nations’ Human Development Index (HDI),^1^ are used to judge the performance of economies.

## 3. A lack of property rights to natural capital

Why do not market prices reflect nature's scarcity value? If natural capital really is becoming scarcer, would not their prices have risen, signalling that all is not well?

The problem is that if prices are to reveal social scarcities, markets must function well. For many types of natural capital, though, most especially ecological resources, markets not only do not function well, often they do not even exist. In some cases, they do not exist because relevant economic interactions take place over large distances, making the costs of negotiation too high (e.g. the effects of upland deforestation on downstream farming and fishing activities; §4); in other cases, they do not exist because the interactions are separated by large temporal distances (e.g. the effect of carbon emission on climate in the distant future, in a world where forward markets do not exist because future generations are not present today to negotiate with us). Then there are cases (the atmosphere, aquifers, the open seas) where the migratory nature of the resource keeps markets from existing—they are called ‘open-access resources’, and they experience the tragedy of the commons.

Each of the above examples points to a failure to have secure *property rights* to natural capital. We can state the problem thus: ill-specified or unprotected property rights prevent markets from forming or make markets function wrongly when they do form.

By ‘property rights’, I do not only mean private property rights, I include communal property rights (e.g. over common property resources, such as woodlands, in South Asia and sub-Saharan Africa) and state property rights. At an extreme end are ‘global property rights’, a concept that is implicit in current discussions on climate change. But the concept is not new. That humanity has collective responsibility over the state of the world's oceans used to be explicit in the 1970s, when politicians claimed that the oceans are a ‘common heritage of mankind’.

The failure to establish secure property rights to natural capital typically means that the services natural capital offers us are underpriced in the market, which is another way of saying that the use of nature's services is implicitly subsidized. At the global level, what is the annual subsidy? One calculation suggested that it is 10 per cent of annual global income (Myers & Kent 2000). My reading is that the margin of error in that estimate is very large. But it is the only global estimate I have come across. Hassan *et al*. (2005) contains quantitative information that could be used to generate more reliable estimates of nature's subsidies. International organizations such as the World Bank have the resources to undertake that work. But they appear to be reluctant to do so.

## 4. Nature's subsidies

Being underpriced, nature is overexploited. So, an economy could enjoy growth in real GDP and improvements in HDI for a long spell even while its overall productive base shrinks. As proposals for estimating the social scarcity prices of natural resources remain contentious, economic accountants ignore them and governments remain wary of doing anything about them. Here is an example of how the use of nature is subsidized.

An easy way for governments to earn revenue in countries that are rich in forests is to issue timber concessions to private firms. Imagine that concessions are awarded in the upland forests of a watershed. Forests stabilize both soil and water flow. So deforestation gives rise to soil erosion and increases fluctuations in water supply downstream. If the law recognizes the rights of those who suffer damage from deforestation, the timber firm would be required to compensate downstream farmers. But compensation is unlikely when (i) the cause of damage is many miles away, (ii) the concession has been awarded by the state,^2^ and (iii) the victims are scattered groups of farmers. Problems are compounded because damages are not uniform across farms: location matters. It can also be that those who are harmed by deforestation do not know the underlying cause of their deteriorating circumstances. As the timber firm is not required to compensate farmers, its operating cost is less than the social cost of deforestation, the latter being the firm's logging costs and the damage suffered by all who are adversely affected. So if the timber is exported abroad, the export contains an implicit subsidy, paid for by people downstream. And I have not included forest inhabitants, who now live under even more straightened circumstances or, worse, are evicted without compensation. The subsidy is hidden from public scrutiny, but it amounts to a transfer of wealth from the exporting to the importing country. Some of the poorest people in a poor country subsidize the incomes of the average importer in what could well be a rich country. That does not feel right.

### (a) Quantifying economic failure

The spatial character of nature's hidden subsidies is self-evident, but getting a quantitative feel involves hard work. So the literature is sparse. As in many other scientific fields, some of the best advances have been made in studies of localized problems. Basing their estimate on a formal hydrological model, Pattanayak & Kramer (2001) reported that the drought mitigation benefits farmers enjoy from upstream forests in a group of Indonesian watersheds are 1–10% of average agricultural incomes. In another paper, Pattanayak & Butry (2005) studied the extent to which upstream forests stabilize soil and water flow in Flores, Indonesia. Downstream benefits were found to be 2–3% of average agricultural incomes.

In a study in Costa Rica on pollination services, Ricketts *et al*. (2004) discovered that forest-based pollinators increase the annual yield in nearby coffee plantations by as much as 20 per cent. Subsequently, Ricketts *et al*. (2008) analysed the results of some two dozen studies, involving 16 crops in five continents, and discovered that the density of pollinators and the rate at which a site is visited by them declines at rapid exponential rates with the site's distance from the pollinators’ habitat. At 0.6 km (respectively, 1.5 km) from the pollinators’ habitat, for example, the visitation rate (respectively, pollinator density) drops to 50 per cent of its maximum.

### (b) Eliminating nature's subsidies

How should societies eliminate nature's subsidies? In the case of the upstream firm and downstream farmers, the state could tax the firm for felling trees. The firm in this case would be the ‘polluter’, the farmers the ‘pollutees’. Pollution taxes are known today as ‘green taxes’. They invoke the *polluter-pays-principle* (PPP). The efficient rate of taxation would be the damage suffered by farmers. What the state does with the tax revenue is a distributional matter, to which I shall return presently.

But there is also a ‘market-friendly’ way to eliminate the subsidies. Lindahl (1958) suggested that the state (or the community) could introduce private property rights to natural capital, the thought being that markets would emerge to price nature's services appropriately. A problem with the proposal, at least as I have presented it here, is that it is not clear who should be awarded property rights. In our example of the upstream firm and downstream farmers, the sense of natural justice might suggest that the rights should be assigned to farmers. Under a system of ‘pollutees-rights’, the timber firm would be required to compensate farmers for the damage it inflicts on them. Such a property-rights regime also invokes PPP.

But the rights could be awarded to the timber firm instead. In that case it would be the farmers who would have to compensate the firm for not felling trees! The latter system of property rights invokes the *pollutee-pays-principle* (a reverse PPP, as it were), which, in the example we are studying, would seem repellent. But it has been argued by proponents that from the efficiency point of view it is a matter of indifference which system of private property rights is introduced.

Market-based systems have attracted much attention among ecologists and development experts in recent years, under the label *payment for ecosystem services* or PES (see Daily & Ellison (2002) and Pagiola *et al*. (2002) for sympathetic reviews of a market-based PES). The ethics underlying PES are seemingly attractive. If decision makers in Brazil believe that decimating the Amazon forests is the true path to economic progress there, should not the rest of the world pay Brazil not to raze them to the ground? If the lake on my farm is a sanctuary for migratory birds, should not bird lovers pay me not to drain it for conversion into farm land? Never mind that the market for ecosystem services could be hard to institute, if a system involving PES were put in place, owners of ecological capital and beneficiaries of ecological services would be forced to negotiate. The former group would then have an incentive to conserve their assets.

Hundreds of new PES schemes have been established round the globe. China, Costa Rica and Mexico, for example, have initiated large-scale programmes in which landowners receive payment for increasing biodiversity conservation, expanding carbon sequestration and improving hydrological services. But although PES may be good for conservation, one can imagine situations where the system would be bad for poverty reduction and distributive justice. Many of the rural poor in poor countries enjoy nature's services from assets they do not own. Even though they may be willing to participate in a system of property rights in which *they* are required to pay for ecological services (Pagiola *et al*. (2008) report in their careful study of a silvo-pastoral project in Nicaragua that they do), it could be that in the world we have come to know, the weaker among the farmers are made to pay a disproportionate amount. Some may even become worse off than they were earlier. One could argue that in those situations the state should pay the resource owner instead, using funds obtained from general taxation. Who should pay depends on the context (Bulte *et al*. 2008).

A PES system in which the state plays an active role is attractive for wildlife conservation and habitat preservation. In poor countries, property rights to grasslands, tropical forests, coastal wetlands, mangroves and coral reefs are often ambiguous. The state may lay claim to the assets (‘public’ property being the customary euphemism), but if the terrain is difficult to monitor, inhabitants will continue to reside there and live off its products. Inhabitants are therefore key stakeholders. Without their engagement, the ecosystems could not be protected. Meanwhile flocks of tourists visit the sites on a regular basis. An obvious thing for the state to do is to tax tourists and use the revenue to pay local inhabitants for protecting their site from poaching and free-riding. Local inhabitants would then have an incentive to develop rules and regulations to protect the site.

## 5. Measuring sustainable development

Whenever economists have probed the matter, they have found that all economies subsidize large numbers of economic transactions with nature. Some of those transactions are large (construction of large dams that alter ecosystems), but mostly they are small. How do those subsidies affect overall economic performance? More fundamentally, how should economic performance be measured?

A famous 1987 report by an international commission (widely known as the Brundtland Commission Report) defined *sustainable development* as ‘ … development that meets the needs of the present without compromising the ability of future generations to meet their own needs’ (World Commission for Environment and Development 1987). In this reckoning, sustainable development requires that relative to their populations each generation should bequeath to its successor at least as large a *productive base* as it had itself inherited. Notice that the requirement is derived from a relatively weak notion of justice among the generations. Sustainable development demands that, relative to population numbers, future generations have no less of the means to meet their needs than we do ourselves; it demands nothing more. But how is a generation to judge whether it is leaving behind an adequate productive base for its successor?

### (a) Shadow prices as social scarcities

We noted earlier that neither GDP nor HDI is of help, because neither is a measure of a country's productive base. So, what does measure the productive base? A society's productive base is the stock of all its capital assets, including its institutions. As we are interested in estimating the change in an economy's productive base over a period of time, we need to know how to combine the changes that take place in its capital stocks.

Intuitively, it is clear that we have to do more than just keep a score of capital assets (so many additional pieces of machinery and equipment, so many more miles of roads, so many fewer square miles of forest cover and so forth). An economy's productive base declines if the decumulation of assets is not compensated by the accumulation of other assets. Contrary-wise, the productive base expands if the decumulation of assets *is* more than compensated by the accumulation of other assets. The ability of an asset to compensate for the decline in some other asset depends on technological knowledge (e.g. double glazing can substitute for central heating up to a point, but only up to a point) and on the quantities of assets the economy happens to have in stock (e.g. the protection trees provide against soil erosion depends on the existing grass cover). The values to be imputed to assets are known as their *shadow prices*. Formally, by an asset's shadow price, we mean the net increase in societal well-being that would be enjoyed if an additional unit of that asset were made available, other things being equal. As shadow prices reflect the social scarcities of capital assets, it is only in exceptional circumstances that they equal market prices.

We are trying to make operational sense here of the concept of *sustainable* development. So we must include in the concept of ‘social well-being’ not only the well-being of those who are alive today, but also of those who will be here in the future. There are ethical theories that go beyond a purely anthropocentric view of nature, by insisting that certain aspects of nature have intrinsic value. The concept of social well-being I am invoking here includes intrinsic values, if that is demanded. However, an ethical theory on its own will not be enough to determine shadow prices, because there would be nothing for the theory to act upon. We need descriptions of states of affairs too. To add a unit of a capital asset to an economy is to perturb that economy. In order to estimate the contribution of that additional unit to societal well-being, we need a description of the state of affairs both before and after the addition has been made, now and in the future. In short, estimating shadow prices involves both evaluation and description.

It should not surprise you that estimating shadow prices is a formidable problem. There are ethical values we hold that are probably impossible to commensurate when they come up against other values that we also hold. That does not mean ethical values do not impose bounds on shadow prices; they do. That is why the language of shadow prices is essential if we wish to avoid making sombre pronouncements about sustainable development that amount to saying nothing. Most methods that are currently deployed to estimate the shadow prices of ecosystem services are crude, but deploying them is a lot better than doing nothing to value them.

### (b) The wealth of nations

The value of an economy's entire stock of capital assets measured in terms of their shadow prices is its *wealth*. Sometimes, we call it *comprehensive wealth*, to remind ourselves that the measure is to include *all* capital assets (building and machinery, roads and rail tracks; health and skills; natural capital and knowledge and institutions), not just reproducible capital such as buildings and machinery, roads and rail tracks. Comprehensive wealth (henceforth, wealth) is a number; expressed, say, in international dollars.

It can be shown that an economy's wealth measures its overall productive base (Hamilton & Clemens 1999; Dasgupta & Mäler 2000; Dasgupta 2001). So, if we wish to determine whether a country's economic development has been sustainable over a period of time, we have to estimate the changes that took place over that period in its wealth relative to growth in population. The theoretical result I am alluding to gives meaning to the title of perhaps the most famous book ever written on economics, namely, *An inquiry into the nature and causes of the wealth of nations*. Observe that Adam Smith did not write about the GDP of nations, nor of the HDI of nations; he wrote about the wealth of nations. It would seem we have come full circle, by identifying sustainable development with the accumulation of (comprehensive) wealth.

### (c) An empirical exercise

In an important paper, Hamilton & Clemens (1999) estimated the change in the wealth of 120 nations during the period 1970–1996 by defining an economy's wealth as the value of its reproducible capital assets and three classes of natural capital assets (commercial forests, oil and minerals and the quality of the atmosphere in terms of its carbon dioxide content). The shadow prices of oil and minerals were taken to be their market prices minus extraction costs. The shadow price of global carbon emission into the atmosphere is the damage caused by bringing about climate change. That damage was taken to be $20 per tonne, which is in all probability a serious underestimate. Forests were valued in terms of their market price minus logging costs. Contributions of forests to ecosystem functions were ignored.

As you can see, the list of natural resources Hamilton and Clemens considered was very incomplete. It did not include water resources, fisheries, air and water pollutants, soil and ecosystems. The authors also ignored improvements in human health and skills, and they did not consider increases in knowledge, nor improvements or deteriorations in the countries’ institutions. Moreover, their estimates of shadow prices were very, very approximate. Nevertheless, one has to start somewhere, and theirs was a first pass at what is an enormously messy enterprise.

In table 1, I offer an assessment of the character of economic development from 1970 to 2000 that is a lot more comprehensive than the one in Hamilton & Clemens (1999). I consider only the poorest regions in the world. I restrict myself to poor countries because I have studied poor countries more than rich countries. I consider Bangladesh, China (a poor country during much of that period), India, Nepal, Pakistan and sub-Saharan Africa. Economists have discovered ingenious ways to estimate the accumulation of knowledge and changes in the effectiveness of an economy's institutions. Those estimates are published regularly by such international organizations as the World Bank. The first column of figures in the table presents my estimates of the average annual percentage rate of change in wealth in each of the regions in the period 1970–2000. My estimates are a refinement of those published by Arrow *et al*. (2004), which in turn were an improvement on those of Hamilton and Clemens: I have added to the Hamilton–Clemens estimates for each region the average annual public expenditure on health and education, the average annual rate of growth in knowledge and changes in the effectiveness of their institutions.

**Table 1.** The progress of poor nations. Adapted from Arrow *et al*. (2004).

	% annual growth rate 1970–2000				
country/region	wealth	population per head	wealth per head	GDP per head	ΔHDI^a^
sub-Saharan Africa	−0.1	2.7	−2.8	−0.1	+
Bangladesh	1.4	2.2	−0.8	1.9	+
India	1.6	2.0	−0.4	3.0	+
Nepal	1.8	2.2	−0.4	1.9	+
Pakistan	1.3	2.7	−1.4	2.2	+
China	5.9	1.4	4.5	7.8	+

^a^Change in HDI between 1970 and 2000.

Notice that, excepting sub-Saharan Africa, wealth increased in every country in my sample. But in judging whether an economy has experienced sustainable development during a period, we have to discover whether wealth has increased *relative to population growth*. The simplest thing to do is to ask whether wealth *per head* has increased. In order to estimate movements in wealth per head, I have collated figures for the average annual population growth rate in each region during the period 1970–2000. They are given in the second column of figures in the table. And in the third column, I present the difference between the figures in the first and second columns, which gives us estimates of the change in wealth per head in each of the regions.

Before summarizing the findings, it will be useful to get a feel for what the table is telling us. Consider Pakistan: during the period 1970–2000 (comprehensive), wealth increased at an average annual rate of 1.3 per cent. But take a look at Pakistan's population, which grew at 2.7 per cent annually. The third column shows that Pakistan's *per capita* wealth *declined* in consequence, at an annual rate of 1.4 per cent, implying that in year 2000 the average Pakistani was a lot poorer than in 1970. Interestingly, if we were to judge Pakistan's economic performance in terms of growth in GDP *per capita*, we would obtain a different picture. As the fourth column of the table shows, Pakistan grew at a respectable 2.2 per cent a year. If we now look at the fifth column, we find that the United Nations’ HDI for Pakistan improved during the period. From 1970 to 2000, Pakistan enjoyed growth in GDP *per capita* and an improvement in HDI by running down its natural capital assets. Movements in GDP *per capita* and HDI tell us nothing about sustainable development.

The striking message of the table is that during the period 1970–2000 economic development in *all* the countries on our list other than China was ‘negative’. To be sure, sub-Saharan Africa offers no surprise. Wealth, not just wealth per head, declined at an annual rate of 0.1 per cent. Population grew at 2.7 per cent a year. Even without performing any calculation, we would have known that the productive base in sub-Saharan Africa declined relative to its population. The table confirms that it did, at 2.8 per cent each year. If we now look at the fourth column of numbers in the table, we discover that GDP *per capita* in sub-Saharan Africa declined at 0.1 per cent annually. But the region's HDI showed an improvement—confirming once again that studying movements in HDI enables us to say nothing about sustainable development.

The table shows that Pakistan is the worst performer in the Indian subcontinent. But the remaining countries in South Asia also did not make it. Admittedly, each country became wealthier, but population growth was sufficiently high to more than neutralize the growth in wealth. Relative to their populations, the productive base in each economy declined. Economic development in South Asia was not sustained.

China was the single exception in my sample. The country invested so much in reproducible capital assets that its wealth grew at an annual rate of 5.9 per cent. Population grew at a relatively low rate: 1.4 per cent per year, which is why China's wealth *per capita* expanded at an annual rate of 4.5 per cent. *Per capita* GDP also grew, at an annual rate of 7.8 per cent and HDI improved. In China, GDP *per capita*, HDI and wealth per head moved parallel to one another.

The figures we have just studied are all *very* rough and ready, but they show how accounting for natural capital can make a substantial difference to our conception of the development process. We should remember that the figures for several shadow prices I used to arrive at the table are conservative. For example, a price of $20 per tonne of carbon in the atmosphere is almost certainly a good deal below its true global social cost. And the methods I have used to value improvements in health and education are almost certainly defective, but in the opposite direction: I have underestimated them. So one of the most important problems we economists face today is to find more effective ways to quantify the progress and regress of nations. So long as we rely on GDP and HDI and the many other ad hoc measures of human well-being, we will continue to paint a misleading picture of economic performance.

Because of their imperfections, the figures in the third column of the table are not be taken literally. Nevertheless, with all the above caveats (and more!) in mind, the overarching moral that emerges from it is salutary:

Development policies that ignore our reliance on natural capital are seriously harmful—they do not pass the mildest test for equity among contemporaries, nor among people separated by time and uncertain contingencies.

## Endnotes

1 HDI is a composite measure of GDP per head, life expectancy at birth and education.

2 Colchester (1995) has recounted that political representatives of forest dwellers in Sarawak, Malaysia, have routinely given logging licenses to members of the state legislature.
